# Scaling up single-cell RNA-seq data analysis with CellBridge workflow

**DOI:** 10.1093/bioinformatics/btad760

**Published:** 2023-12-19

**Authors:** Nima Nouri, Andre H Kurlovs, Giorgio Gaglia, Emanuele de Rinaldis, Virginia Savova

**Affiliations:** Precision Medicine and Computational Biology, Sanofi, Cambridge, MA 02141, United States; Precision Medicine and Computational Biology, Sanofi, Cambridge, MA 02141, United States; Precision Medicine and Computational Biology, Sanofi, Cambridge, MA 02141, United States; Precision Medicine and Computational Biology, Sanofi, Cambridge, MA 02141, United States; Precision Medicine and Computational Biology, Sanofi, Cambridge, MA 02141, United States

## Abstract

**Summary:**

Single-cell RNA sequencing (scRNA-seq) has revolutionized the study of gene expression at the individual cell level, unraveling unprecedented insights into cellular heterogeneity. However, the analysis of scRNA-seq data remains a challenging and time-consuming task, often demanding advanced computational expertise, rendering it impractical for high-volume environments and applications. We present CellBridge, an automated workflow designed to simplify the standard procedures entailed in scRNA-seq data analysis, eliminating the need for specialized computational expertise. CellBridge utilizes state-of-the-art computational methods, integrating a range of advanced functionalities, covering the entire process from raw unaligned sequencing reads to cell type annotation. Hence, CellBridge accelerates the pace of discovery by seamlessly enabling insights into vast volumes of scRNA-seq data, without compromising workflow control and reproducibility.

**Availability and implementation:**

The source code, detailed documentation, and materials required to reproduce the results are available on GitHub and archived in Zenodo. For the CellBridge pre-processing step (v1.0.0), access the GitHub repository at https://github.com/Sanofi-Public/PMCB-ToBridge and the Zenodo archive at https://zenodo.org/records/10246161. For the CellBridge processing step (v1.0.0), visit the GitHub repository at https://github.com/Sanofi-Public/PMCB-CellBridge and the Zenodo archive at https://zenodo.org/records/10246046.

## 1 Introduction

Recent advancements in machine learning and artificial intelligence technologies have presented exciting opportunities for leveraging single-cell transcriptomic data to optimize analytical models and identify those with the highest predictive potential for therapeutic applications (Gigante *et al.* 2022, Xu *et al.* 2023). The primary driver behind the success of such models lies in the availability of abundant and reliable training data, enabling the discovery of meaningful patterns and accurate predictions. Despite the exponential growth in scRNA-seq data volume due to the democratization of sequencing technologies, the conventional manual processing approach remains prevalent in scientific settings. Unfortunately, this manual approach is prone to errors and vulnerable to investigator bias, as different avenues of data analysis may lead to non-predictive models or biased outcomes. Consequently, there is an urgent need to streamline the analysis of scRNA-seq data through the automation of robust and reproducible workflows, aiming to effortlessly generate an unbiased portfolio of scRNA-seq data.

The application of scRNA-seq analysis in industrial settings has gained traction due to its increasing value in generating disease and drug-related insights (Van de Sande *et al.* 2023). However, ensuring the reproducibility of results in this context presents a critical challenge. Variability can arise from the utilization of diverse computing environments and software, thereby affecting the consistency of outcomes. The improvisation-driven, free-flowing analytics often employed in academic settings are not easily adaptable to clinical studies and regulated environments like good practice (GxP) for experimental settings. In these contexts, strict adherence to controlled timelines and reliable outputs is imperative. The standard approach to scRNA-seq analysis poses an additional obstacle, particularly for researchers lacking the necessary training. This limitation hinders the accessibility of scRNA-seq analysis, impacting both academic and industrial environments.

Numerous scRNA-seq analysis workflows have been developed to address the challenges associated with analyzing scRNA-seq data (Chen *et al.* 2019, Luecken and Theis 2019, Amezquita *et al.* 2020). However, the wealth of biological insights embedded in scRNA-seq data has led to a constant influx of new computational tools, creating a challenge for investigators to stay abreast of the latest advancements in this rapidly evolving landscape of tools and analysis steps. Many existing workflows for scRNA-seq data processing are either no longer actively maintained or lack essential steps necessary for comprehensive scRNA-seq analysis, such as the conversion of sequencer binary base call (BCL) files into human-readable text format (FASTQ files) or read alignment (Grandi *et al.* 2022, Prieto *et al.* 2022, Rich-Griffin *et al.* 2023), as well as robust cell type identification (Garcia-Jimeno *et al.* 2022). Furthermore, the computational complexity associated with most available workflows often presents a significant barrier, restricting their usage to investigators possessing advanced computational skills (Rich-Griffin *et al.* 2023). Consequently, none of the existing workflows fully satisfy the requirements of fast-paced high-volume scientific environments, where large quantities of data are generated on a daily basis and demand rapid and consistent processing.

We introduce CellBridge, a user-friendly workflow designed to tackle the challenges of analyzing scRNA-seq data. CellBridge offers a comprehensive end-to-end ecosystem, starting from unaligned sequencer reads and leading to refined data objects, as well as an illuminating HTML summary report. The streamlined design of CellBridge obviates the need for extensive programming skills, opening the doors of scRNA-seq analysis to non-computational investigators (Supplementary Fig. S1). We showcase the application of CellBridge in processing a published scRNA-seq dataset from patients with chronic obstructive pulmonary disease. By streamlining the analysis of scRNA-seq data, our goal is to expedite the discovery of valuable biological insights, empowering the wider scientific community to fully harness the potential of this formidable technology.

## 2 Methods

### 2.1 CellBridge workflow overview

CellBridge is a Docker-based pipeline that consists of a series of functions written in R and Python, which are called sequentially. Each function in the pipeline performs a specific task, and the functions are designed to take input data from the previous function, process it, and produce output data for the next function in the pipeline (Supplementary Fig. S2).

The pipeline is divided into two main sub-workflows: pre-processing (Supplementary Fig. S2A) and processing (Supplementary Fig. S2B). The first workflow carries out converting sequencer output files (BCL files) into a human-readable text format (FASTQ files), performing reads quality control, and conducting read alignment and gene expression quantification. Once the data has undergone pre-processing, it is then passed on to the second workflow for processing. The processing workflow encompasses standard steps for scRNA-seq analysis, including quality control, doublet removal, normalization, batch correction, dimensionality reduction, clustering, identification of cell markers, and cell-type annotation.

### 2.2 CellBridge pre-processing step

The pre-processing workflow accepts both BCL (binary base call) and FASTQ (text-based sequence file format) files. Depending on the input data type, different paths are followed (Supplementary Fig. S2A). The user can provide the raw data in BCL format generated by the sequencing instrument. In this case, the pre-processing workflow starts with base calling to convert the raw data into FASTQ format. If the user has already demultiplexed the raw data into FASTQ files, they can directly provide those files to the pre-processing workflow. In this case, the workflow will skip the base calling step and start from the processing of the FASTQ files.

For the base calling step, two current state-of-the-art methods, Cell Ranger mkfastq (www.10xgenomics.com/support) and BCL Convert (www.support.illumina.com), have been implemented into the pre-processing workflow. This gives users the flexibility to choose between the two tools based on their preference or the sequencing platform they used. Regardless of the tool used, the output FASTQ files will be subsequently processed by the workflow for further analysis. It is worthy to note that BCL Convert offers improvements in the speed and efficiency of handling large data sets compared to the older bcl2fastq (www.support.illumina.com), which is the core engine of the Cell Ranger mkfastq method.

The quality control step in the pre-processing workflow involves the sequence analysis tool, FastQC (www.bioinformatics.babraham.ac.uk). FastQC is a free, open-source software that analyzes FASTQ files to assess their quality and provide users with a summary report. The quality control step can identify technical biases or issues in the sequencing data, such as read quality, GC content, sequence length distribution, or adapter contamination. These issues can impact downstream analysis, so it is important to identify and address them before proceeding with further analysis.

The pre-processing workflow provides users with two options for the alignment and quantification step: Cell Ranger count (www.10xgenomics.com/support) and STARsolo (Kaminow *et al.* 2021). Both methods are widely used in the field and therefore have been implemented in the workflow to offer flexibility in the choice of alignment and quantification tools. The selection of a specific method can depend on factors such as data type, sequencing depth, and downstream analysis goals. We note that both Cell Ranger count and STARsolo use the STAR algorithm (Dobin *et al.* 2013) which performs splicing-aware alignment of reads to the genome. Regardless of the choice, the output files generated by the alignment and quantification step will be transformed into a format compatible with downstream processing workflow, facilitating the seamless integration of the data into the subsequent analytical pipeline.

### 2.3 CellBridge processing step

The CellBridge processing pipeline is a Seurat-based workflow that performs a comprehensive range of scRNA-seq data analysis tasks including quality control, doublet removal, normalization, batch correction, dimensionality reduction, clustering, and identification of cell markers using cutting-edge methodologies (Supplementary Fig. S2B). Cell-type annotation is another critical step in the analysis of scRNA-seq data, as it enables the identification and characterization of distinct cell populations. To facilitate this process, the CellBridge workflow incorporates two cell type annotation methodologies, Sargent (Nouri *et al.* 2023) and SignacX (Chamberlain *et al.* 2023), which were developed by the authors and published previously. SignacX is a pre-trained classifier designed to predict cellular phenotypes in scRNA-seq data based on the Human Primary Cell Atlas. Sargent is a score-based method that predicts the cell type of origin based on a set of marker genes associated with each cell type. By integrating these two annotation methodologies into the CellBridge workflow, investigators can efficiently and comprehensively gain insights into the biology of their scRNA-seq data.

CellBridge accepts different types of input data for analysis. The first type is the widely used output of the 10X-Genomics Cell Ranger pipeline: the trio of the matrix of UMI counts, the list of cell barcodes, and the list of gene names. Additionally, CellBridge accepts Hierarchical Data Format (HDF5, h5) file formats that are generated by the Cell Ranger pipeline. CellBridge also accepts count matrix files in txt.gz format, which contain the scRNA-seq gene expression quantification information. The feature-barcode matrices can be generated by any microfluidic-, microwell plates-, or droplet-based scRNA-seq technology. Finally, CellBridge accepts previously processed Seurat RDS (R Data Serialization) objects as the input. Regardless of the input data type, CellBridge transforms the data into a format that is compatible with downstream analyses. The CellBridge processing step is an independent entity from the pre-processing step, offering investigators the option to utilize it when count matrices are already available.

CellBridge generates three output files. The first file is a comprehensive summary HTML file that serves as a quick reference guide and snapshot of the analysis outcomes from the pipeline. The second file is an RDS format file containing all the data required to reproduce the HTML file, facilitating easy sharing and review of the analysis with others. The third file is an RDS format file that includes a Seurat object with both the provided metadata and computed attributes. The trio of output files empowers investigators to further explore and analyze their scRNA-seq data as desired.

## 3 Results

We selected the publicly available dataset PRJEB44878 (Wohnhaas *et al.* 2021) as a use case to demonstrate CellBridge’s capabilities. The dataset consists of primary small airway epithelial cells (SAECs) isolated from the lungs of healthy controls (*n* = 3) and chronic obstructive pulmonary disease (COPD) patients (*n* = 3). The SAECs were expanded in vitro and differentiated into a pseudostratified epithelium under air–liquid interface (ALI) conditions. To simulate smoke-induced injuries to the small airways of healthy non-smokers and COPD smokers, the fully differentiated SAEC ALI cultures were exposed either to whole cigarette smoke for four consecutive days or to ambient air as control. We assessed the performance of the CellBridge workflow using this dataset. The results are displayed in the HTML report summary, Supplementary Material S1, which is accompanied by a detailed description of each section of the report in Supplementary Material S2.

## 4 Discussion

Over the past decade, the field of single-cell transcriptomics has undergone a remarkable transformation, evolving from a highly specialized assay into a commercially accessible technology with widespread adoption. Simultaneously, software developers have created an extensive array of algorithms to process data, analyze outputs, and extract biomedical insights. Currently, the organization of analytics predominantly relies on shared computing platforms, with Seurat (Satija *et al.* 2015, Hao *et al.* 2021) and Scanpy (Wolf *et al.* 2018, Sturm *et al.* 2020) emerging as the most extensively utilized tools. However, these platforms demand expertise in computational biology to effectively analyze data and develop novel code. Nevertheless, with the democratization of both experimental and analytical workflows and the surge in data volume, there is a growing need for unified pipelines that empower investigators without advanced computational degrees to accurately analyze scRNA-seq experiments. Additionally, as single-cell technologies find their way into the clinical trials ecosystem, the importance of reproducibility, data privacy, and regulatory compliance cannot be understated, yet these aspects remain largely unaddressed by current workflows. In this study, we present the CellBridge pipeline as a secure solution designed to expand the utilization of single-cell analytics to a wider range of biomedical investigators, regardless of their computational skills, in both exploratory and clinical settings.

CellBridge is constructed upon a Docker-based foundation, providing a robust and reproducible environment for seamless execution of the pipeline. This Docker image can be transferred between diverse execution environments (push-to-registry and pull-to-host), ensuring portability and compatibility of the pipeline. Additionally, CellBridge provides a user-friendly command-line interface that, in conjunction with the Docker-based foundation, facilitates the execution of the workflow in computing environments that adhere to guidelines and regulations governing clinical data. Furthermore, CellBridge provides a comprehensive report of the technical computing specifications utilized during processing. This transparency is a fundamental requirement for scientific research and the advancement of clinical applications.

In conclusion, by facilitating the transparent and reproducible analysis of scRNA-seq data on computational platforms that adhere to stringent data protection regulations, the CellBridge workflow possesses the potential to significantly impact both forward- and backward-translation in clinical research. The CellBridge project is continually evolving, with ongoing developments such as new modules, advanced analyses, and improved visualization features.

## Supplementary Material

btad760_Supplementary_Data

## Data Availability

The scRNA-seq data used for a showcase are available in the European Nucleotide Archive database under accession code PRJEB44878.
