# Supplementary material for: Scaling up single-cell RNA-seq data analysis with CellBridge workflow
Source: Bioinformatics. 2023 Dec 19;39(12):btad760. doi: 10.1093/bioinformatics/btad760 (PMC10751228; doi:10.1093/bioinformatics/btad760)
Supplement: btad760_Supplementary_Data [file btad760_supplementary_data.zip › Supplementary_Material_S2.pdf]

Scaling up Single-Cell RNA-seq Data Analysis with CellBridge Workflow

Nima Nouri[*,†,1], Andre H. Kurlovs[†,1], Giorgio Gaglia[1], Emanuele de Rinaldis[1], Virginia Savova[*,1]

[1]Precision Medicine and Computational Biology, Sanofi, 350 Water Street, Cambridge, MA 02141, USA

[†]Authors contributed equally.

*To whom correspondence should be addressed.
Contact: nima.nouri@sanofi.com and virginia.savova@sanofi.com

Table of Contents

1 Results *1*

1.1 CellBridge processed data summary 2

1.2 CellBridge workflow settings 3

1.3 Sample-level characteristics and quality control metrics 3

1.4 Cell clusters composition 5

1.5 Cell type annotation 6

1.6 Trajectory inference analysis 6

1.7 Data source and reference 7

1.8 Specifics of the computing environment 7

References *7*

Supplementary Figure S1 ..... *9*

Supplementary Figure S2 ..... *10*

Supplementary Figure S3 ..... *11*

1 Results

FASTQ files are provided for each patient group (n=12) in the European Nucleotide Archive database under accession code PRJEB44878 (Wohnhaas 2021). We used pre-processing `cellranger-count` function to align the reads and generate count matrices for each sample. The output count matrices are automatically organized into a nested directory structure (one folder per sample) to ensure compatibility with the CellBridge processing step. To guide the processing workflow regarding data loading, a

metadata file was provided alongside the count matrices for the process. The metadata file, which is constructed based on Table S3 from the published manuscript, is available on CellBridge GitHub. The workflow processed over 40,000 cells from 12 samples in a 3-hour run on an AWS r5.4xlarge (1024GB) processor, resulting in the generation of the trio of outputs. Following, we provided a detailed description of each section of the HTML report summary (**Supplementary Fig. S3 and Supplementary Material S1**).

The first component of the HTML report is the header, which includes the project title, unique identifier, and date. The project title is provided by the user during workflow setup to ensure easy identification of the specific project. CellBridge generates a unique identifier (UID) for all three output files. The UID is a 15-character alphanumeric code (consisting of upper and lower-case letters and numbers) that is tagged to all three output file names and projected on the HTML summary report. The UID serves as a tracking mechanism for the data in case the same dataset is processed multiple times with different input parameters. The UID ensures that the output files can be easily identified and distinguished, allowing investigators to easily trace their analysis and results back to the specific run and set of parameters used and minimizing confusion and errors in data management. The remaining of the HTML report is comprised of several components focused on quality control, cell population analysis, and cell-type annotation. The report encompasses 8 main sections: (1) Data Summary, (2) BridgeSettings, (3) BridgeQC, (4) BridgeCluster, (5) BridgeAnnotation, (6) BridgeTrajectory, (7) References, and (8) Session Information (**Supplementary Fig. S3 and Supplementary Material S1**).

1.1 CellBridge processed data summary

Section 1 provides a quantitative summary of the input and processed data, including the total number of input cells and the final number of cells after quality control (QC). Additionally, this section reports the average number of unique molecular identifiers (UMIs) and genes per cell post-QC. In our analysis of the PRJEB44878 raw data, after applying QC filters to the dataset, a total of 34,200 cells were retained, resulting in a loss of approximately 15.0% of the original 40,178 cells. Moreover, the final object has an average of 14,425 UMIs and 3,802 genes per cell (**Supplementary Material S1 – Section 1**). These results provide insights into the quality of the dataset and demonstrate the impact of the QC steps performed by the workflow.

1.2 CellBridge workflow settings

Section 2 displays the configuration settings for the workflow used in the analysis. This section provides a list of all the parameters used during the analysis, ensuring transparency in the data processing steps. For the use case, we utilized common QC parameters such as the minimum number of UMIs per cell, set to 750, minimum number of genes per cell, set to 250, minimum number of cells expressing a given gene, set to 3, percentage of mitochondrial genes per cell, set to 15, and the threshold used for doublet identification, set to 0.25. A full list of settings used in this analysis can be found in **Supplementary Material S1 – Section 2**.

1.3 Sample-level characteristics and quality control metrics

Section 3 is dedicated to presenting deeper information on the quality control measures (**Supplementary Material S1 – Section 3**). Section 3.1 is divided into five tabs that provide an exhaustive sample-wise summary of metadata, demographics, and QC metrics. The first tab displays metadata and demographic information for each sample (**Supplementary Material S1 – Section 3.1.1**), while the second tab reports the number of cells and genes pre- and post-QC for each sample (**Supplementary Material S1 – Section 3.1.2**). The third, fourth, and fifth tabs present the number of UMIs, genes, and the percentage of mitochondrial genes per cell in a quantile format (i.e., divided into quarters, with each quarter representing 25% of the data distribution) post-QC, respectively, for each sample (**Supplementary Material S1 – Sections 3.1.3-5**).

To facilitate visual assessment of the key quality control metrics, Section 3.2 includes three tabs plotting the distribution of UMIs, genes, and percentage of mitochondrial genes expression per cell for each sample, enabling detection of potential outlier samples. In addition, the QC thresholds set by the user are represented by dashed lines, providing a clear indication of which cells meet the established quality criteria. In the PRJEB44878 use case, the majority of cells passed the QC thresholds, and those falling beyond the QC thresholds were excluded from further analysis (**Supplementary Material S1 – Sections 3.2.1-3**).

Section 3.3 consists of two tabs to facilitate the assessment of barcode contamination in multi-sample experiments. Tab 1 allows for pairwise comparisons of samples, quantifying overlapping barcodes, while Tab 2 displays the corresponding percentages after QC filtering. By identifying and measuring barcode overlap, investigators can assess the degree of contamination, if any, and take measures to improve the accuracy and reliability of their data. In the PRJEB44878 use case, we observed only a small number of

overlapping barcodes (<1%) in each pairwise comparison, indicating minimal contamination during sample preparation (**Supplementary Material S1 – Sections 3.3.1-2**).

Section 3.4 is dedicated to the removal of doublet cells. This section includes two tabs that display the distribution of doublet scores calculated using the Scrublet package (Wolock 2019). The first tab presents observed scores, while the second tab shows simulated scores. Both tabs include a dashed line representing the threshold used to identify cells with high doublet scores. In the PRJEB44878 use case, we observed an upper long tail in the distribution of observed scores, which was truncated after applying a 0.25 cut-off threshold, the default value in Scrublet vignette (**Supplementary Material S1 – Sections 3.4.1-2**).

Section 3.5 consists of two tabs and is primarily intended to evaluate the impact of quality control on cell, gene, and UMI abundances in the dataset. Tab 1 compares the number of UMIs and genes before and after QC for each sample, while Tab 2 displays the average number of UMIs and genes per sample after QC. These plots provide a visual representation of the changes in cell and gene counts resulting from QC, allowing investigators to evaluate the effectiveness of their QC strategy. Analysis of the PRJEB44878 dataset revealed a consistent impact of QC filtering on all samples with a similar pattern of change (**Supplementary Material S1 – Sections 3.5.1**), and all samples exhibited a comparable average number of UMIs and genes (**Supplementary Material S1 – Sections 3.5.2**), indicating a uniform QC impact across samples.

In section 3.6, the final object resulting from the merging of post-QC samples (n=12; one Seurat object per each sample) is examined, and key QC metrics (UMI count, gene count, and mitochondrial expression) calculated per cell are displayed in a quantile format (**Supplementary Material S1 – Section 3.6**). Next, Section 3.7 is dedicated to the visualization of the merged object using dimensionality reduction techniques (2D-manifolds). This section comprises three tabs presenting the cells in SPRING (a force-directed graph layout algorithm; (Weinreb 2018)), UMAP (uniform manifold approximation and projection), and TSNE (t-distributed stochastic neighbor embedding) spaces. Such visualizations are crucial for scRNA-seq data analysis as they provide unique insights into the cellular composition of each sample. For instance, UMAP is effective in maintaining global structure, whereas t-SNE is better suited for separating out local clusters, and SPRING highlights community density structures. Two extra tabs have also been implemented to visualize the dataset's status before batch correction on 2D-manifolds UMAP and TSNE. These tabs allow an examination of the before-and-after effects of batch removal in case of batch correction usage. In the processed PRJEB44878 dataset, we used Harmony (Korsunsky

2019) to remove batch effects. We observed consistent overlap of samples across all three visualization techniques suggesting that the processed data is free from batch effects (**Supplementary Material S1 – Sections 3.7.1-3**).

Finally, section 3.8 utilizes the 2D-manifolds to display the key post-QC metrics for each cell. This provides investigators with the ability to identify specific cell populations that differ substantially in quality compared to other populations. In the processed PRJEB44878 dataset, we observed a uniform distribution of key post-QC metrics across all cells, indicating no unexpected transcriptomic bias in the subpopulations (**Supplementary Material S1 – Sections 3.8.1-9**).

1.4 Cell clusters composition

After thoroughly assessing the quality of the samples, our focus shifts to Section 4, which is dedicated to clustering cell populations. In Section 4.1, we utilized the 2D-manifolds to display color-coded populations of cells. CellBridge uses two different approaches to perform clustering. First, according to the default Seurat strategy and the second based on the implementation in the SignacX package (Chamberlain 2023). By comparing the results of both clustering methods, researchers can assess the robustness of the cell population identifications and evaluate the potential impact of methodological differences. Analysis of the PRJEB44878 dataset using both methods revealed 18 clusters (**Supplementary Material S1 – Sections 4.1.1-3**).

Section 4.2 delves into the abundances and relative proportions of the identified cell populations, providing an in-depth look at the prevalence of each cell population (**Supplementary Material S1 – Sections 4.2.1-2**). Next, section 4.3 provides a more granular understanding of the distribution of cells within each cluster. We evaluate the statistical dispersion across samples within each identified cluster by calculating the Gini index. The Gini index capture the extent of variability across samples. For instance, a high Gini index (larger than 0.5) indicates that the cluster is dominated by a single or a small group of samples. The observed bias may be indicative of batch artifacts present in the data or may suggest an underlying biological characteristic. The resulting values from PRJEB44878 are shown in a scatterplot, sorted from largest to smallest cluster, to allow for a visual identification of clusters with high Gini index and hence a sample prevalence bias (**Supplementary Material S1 – Sections 4.3.1-2**).

Section 4.4 utilizes differential expression analysis to identify genes that are significantly upregulated in each cluster compared to the rest of the cells. This involves comparing the expression of each gene in

the cells within a given cluster to that of the remaining cells, with a p-value adjustment of less than 0.05 to identify the top significantly expressed genes (Seurat's default implementation of the Wilcoxon rank-sum test). The resulting gene list provides a deeper understanding of the transcriptomic profile of each cluster, aiding in the identification of potential biomarkers for further investigation (**Supplementary Material S1 – Sections 4.4.1-2**).

1.5 Cell type annotation

Section 5 is dedicated to cell type annotation results. In section 5.1, the 2D-manifolds are employed to display color-coded cell type populations, identified by SignacX and Sargent. In Section 5.2, the abundances and relative proportions of identified cell types are presented. Section 5.3, designated for Sargent, presents a tooltip network infographic that visualizes the hierarchy of the user-provided gene set. Each node represents a specific cell type and displays a pop-up window listing the user-defined marker genes. This visualization aids investigators in the interpretation of the cell type annotation outcomes. Section 5.4 is dedicated to Sargent as well, which visualizes the expression levels of user-provided gene markers across identified cell types, following the given cell type ontology hierarchy. This visualization allows investigators to assess the quality of the cell type annotation outcome by inspecting the expression levels of the assigned gene markers.

Our analysis of the PRJEB44878 dataset using both annotation methods, SignacX and Sargent, revealed high concordance between them in the identification of all cells as epithelial, which is expected since the samples were differentiated from primary small airway epithelial cells. This highlights the consistent performance of the two incorporated methods in accurately identifying the main cell type present in the dataset. However, for a more detailed characterization of the epithelial cell population, Sargent was able to identify distinct cell types, including 15,097 MUC5AC+ goblet cells (44%), 9,626 FOXJ1+ ciliated cells (28%), 9,063 KRT5+ basal cells (26%), and 342 SCGB1A1+ club cells (1%). In addition, Sargent identified rare epithelial cells such as 28 aberrant KRT17+ basaloid cells (<1%), 22 FOXI1+ ionocyte cells (<1%), and 22 GRP+ pulmonary neuroendocrine cells (PNECs) (<1%) (**Supplementary Material S1 – Sections 5.1-4**). These findings offer valuable insights into the cellular landscape of the studied biological system and highlight the efficacy of the workflow for identifying both common and rare cell types at various levels of granularity. The gene set used for the analysis is available in CellBridge GitHub.

1.6 Trajectory inference analysis

Section 6 is dedicated to trajectory inference analysis, which is particularly valuable for studying processes such as cell differentiation, development, or responses to perturbations. Trajectory inference results are often visualized using pseudo-temporal ordering plots or trajectory plots, providing insights into the dynamics of cellular states over a continuum. In Section 6.1, we utilize the Slingshot algorithm (Street 2018) to reconstruct the developmental trajectories of individual cells across inferred Seurat clusters. This section comprises two tabs presenting the inferred trajectories on 2D-manifolds UMAP and TSNE (**Supplementary Material S1 – Sections 6.1.1-2**). In section 6.2, we have incorporated an interactive visualization that effectively displays the pseudotime progression of cells across different lineages, grouped by their respective clusters and corresponding manifolds UMAP and TSNE (**Supplementary Material S1 – Sections 6.2.1-2**). This visualization offers a more dynamic and informative representation of the inferred trajectories. In Section 6.3, we extend our analysis by conducting a thorough examination to identify and report the top differentially expressed genes along each trajectory (**Supplementary Material S1 – Sections 6.3.1-2**). Utilizing this method provides a granular perspective on the shifts in gene expression linked to the cells' developmental pathways.

1.7 Data source and reference

Section 7 provides two hyperlinked placeholders that allow users to access the references for the published paper and related data of the project. This section is a helpful resource for investigators to easily access the given references and further explore the related metadata and findings presented in the published paper (**Supplementary Material S1 – Section 7**).

1.8 Specifics of the computing environment

The final section of the report, Section 8, provides a comprehensive list of the technical specifications used during CellBridge processing, including the versions of all the packages, libraries, and dependencies utilized (**Supplementary Material S1 – Section 8**). The documentation of such information is a critical resource to reproduce the workflow and validate the results presented in the report.

References

Chamberlain, Mathew and Nouri, Nima and Kurlovs, Andre and Hanamsagar, Richa and Nestle, Frank O and de Rinaldis, Emanuele and Savova, Virginia. 2023. "Cell Type Classification and Discovery across Diseases, Technologies and Tissues Reveals Conserved Gene Signatures of Immune Phenotypes." *Journal of Bioinformatics and Systems Biology* (Fortune Journals) 6 (3): 152.

Korsunsky, Ilya and Millard, Nghia and Fan, Jean and Slowikowski, Kamil and Zhang, Fan and Wei, Kevin and Baglaenko, Yuriy and Brenner, Michael and Loh, Po-ru and Raychaudhuri, Soumya. 2019. "Fast, sensitive and accurate integration of single-cell data with Harmony." *Nature methods* (Nature Publishing Group US New York) 16 (12): 1289--1296.

Street, Kelly and Risso, Davide and Fletcher, Russell B and Das, Diya and Ngai, John and Yosef, Nir and Purdom, Elizabeth and Dudoit, Sandrine. 2018. "Slingshot: cell lineage and pseudotime inference for single-cell transcriptomics." *BMC genomics* (Springer) 19: 1--16.

Weinreb, Caleb and Wolock, Samuel and Klein, Allon M. 2018. "SPRING: a kinetic interface for visualizing high dimensional single-cell expression data." *Bioinformatics* (Oxford University Press) 34 (7): 1246--1248.

Wohnhaas, Christian T and Gindele, Julia A and Kiechle, Tobias and Shen, Yang and Leparc, Germ{\'a}n G and Stierstorfer, Birgit and Stahl, Heiko and Gantner, Florian and Viollet, Coralie and Schymeinsky, J{\"u}rgen and others. 2021. "Cigarette Smoke specifically affects small airway epithelial cell populations and triggers the expansion of inflammatory and squamous differentiation associated basal cells." *International Journal of Molecular Sciences* (MDPI) 22 (14): 7646.

Wolock, Samuel L and Lopez, Romain and Klein, Allon M. 2019. "Scrublet: computational identification of cell doublets in single-cell transcriptomic data." *Cell systems* (Elsevier) 8 (4): 281--291.

Supplementary Figure S1

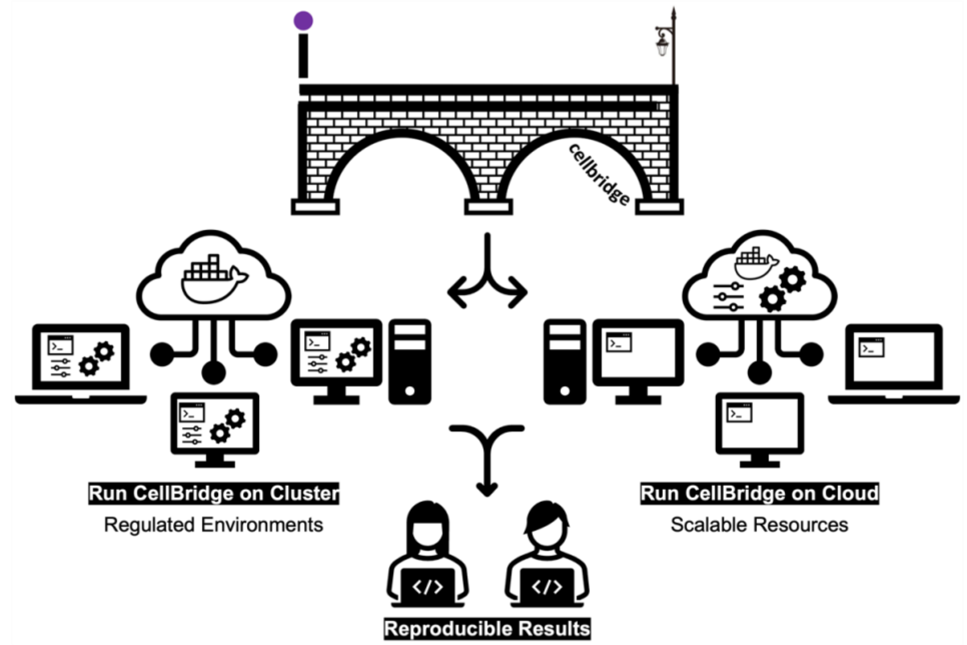

Schematic representation of the CellBridge workflow execution. *CellBridge offers a user-friendly and accessible docker-based workflow that enables reproducible analysis outcomes, regardless of the computing environment in use.*

Supplementary Figure S2

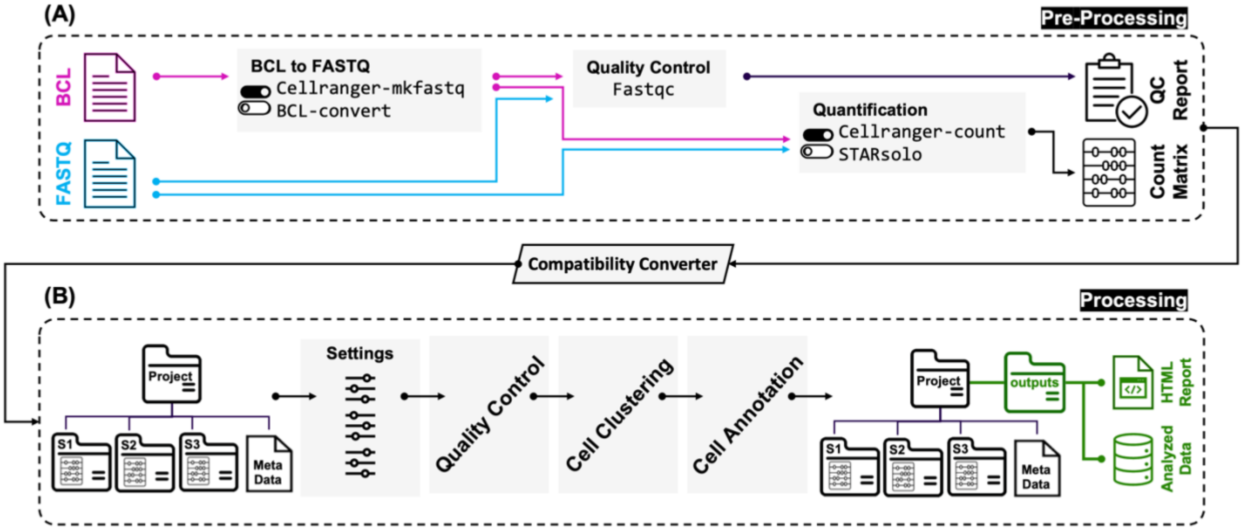

CellBridge workflow overview. *CellBridge is a fully automated workflow that provides end-to-end analysis of scRNA-seq data. It begins with the preprocessing of unaligned sequencer outputs (A) and concludes with the execution of complex downstream analysis (B). The workflow is designed to be compatible with both sequencer output BCL files (panel A - magenta path) and human-readable text format FASTQ files (panel A - blue path). BCL files can be converted to FASTQ format using cellranger-mkfastq or BCL-convert. Regardless of the specific tool used, the output FASTQ files are processed by the workflow for quality control using FastQC, and for alignment and quantification using Cell Ranger-Count or STARsolo. Users can input demographic and clinical information for each sample or cell by utilizing a metadata file, either sample-based or cell-based, respectively. The metadata file must include a column that contains the name of each sample. The sample names specified in the metadata file should match the names of the directories containing the scRNA-seq gene expression matrices for each sample. This correspondence between sample names and directory names is necessary for workflow to load the data and correctly associate the metadata with each sample during the analysis process. Each input dataset should be placed in a separate directory named after the corresponding sample. If users are utilizing the pre-processing step, the required directory organization will be automatically handled by the compatibility converter integrated into the workflow.*

Supplementary Figure S3

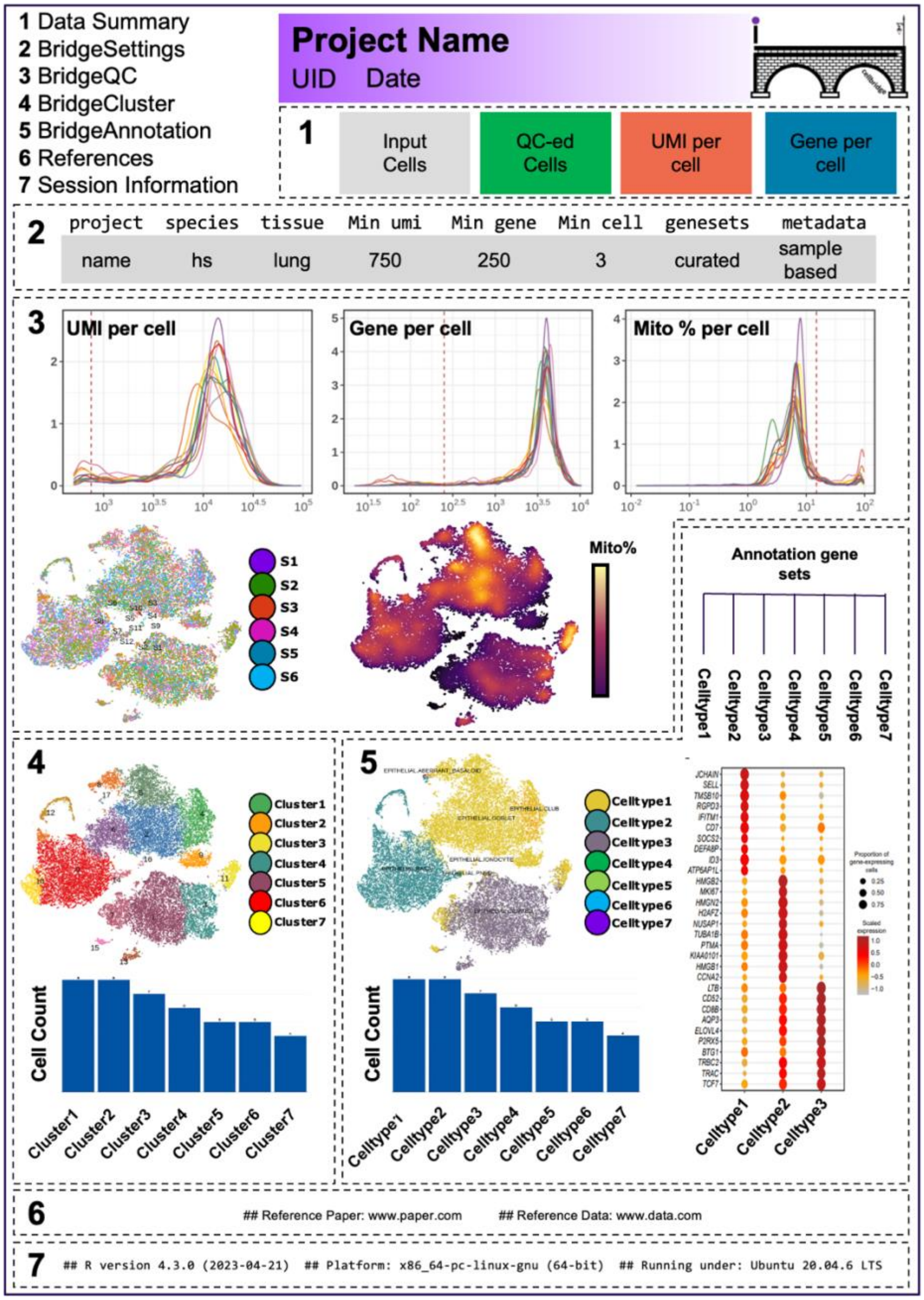

***Illustrative representation of the HTML report generated by the CellBridge workflow. Panel 1** showcases an illustrative representation of the Data Summary section, offering a quantitative overview of the input and processed data. **Panel 2** showcases an illustrative representation of the BridgeSettings section, which reports the configuration settings employed in the analysis workflow. **Panel 3** showcases an illustrative representation of the BridgeQC section, providing an exhaustive analysis of scRNA-seq related quality control measurements. **Panel 4** showcases an illustrative representation of the BridgeCluster section, focusing on clustering cell populations. **Panel 5** showcases an illustrative representation of the BridgeAnnotation section, focusing on cell type annotation. **Panel 6** showcases an illustrative representation of the References section, providing access to the published paper and data. **Panel 7** showcases an illustrative representation of the Session Information section, offering details about the session and environment settings. To explore a full CellBridge-generated HTML report, see **Supplementary Material S1**.*
