# Supplementary material for: Scaling up single-cell RNA-seq data analysis with CellBridge workflow
Source: Bioinformatics. 2023 Dec 19;39(12):btad760. doi: 10.1093/bioinformatics/btad760 (PMC10751228; doi:10.1093/bioinformatics/btad760)
Supplement: btad760_Supplementary_Data [file btad760_supplementary_data.zip › Supplementary_Material_S1.html]

COPD-PRJEB44878


# COPD-PRJEB44878

#### Identifier: cellbridge\_v1.0.0\_ZaZNFm7oklE2GzR

#### Date: 26 November, 2023


---

# 1 Data Summary

---

# 2 BridgeSettings

Configuration Settings for the Workflow

| species | tissue | metadata | genesets | genetype | min\_umi\_per\_cell | max\_mt\_percent | min\_genes\_per\_cell | min\_cell | scr\_th | seu\_nrmlz\_method | seu\_scale\_factor | seu\_n\_hvg | seu\_n\_dim | seu\_k\_param | seu\_cluster\_res | harmony | tsne | spr\_n\_dim | mrk\_logfc | mrk\_min\_pct | mrk\_only\_pos | mrk\_test | mrk\_top\_n | adt | trajectory | traj\_var\_gene | traj\_top\_n | sargent |
| --- | --- | --- | --- | --- | --- | --- | --- | --- | --- | --- | --- | --- | --- | --- | --- | --- | --- | --- | --- | --- | --- | --- | --- | --- | --- | --- | --- | --- |
| hs | lung | sample\_based | curated | none | 750 | 15 | 250 | 3 | 0.25 | LogNormalize | 1e+06 | 2000 | 30 | 20 | 0.7 | sample | TRUE | 30 | 0.25 | 0.5 | TRUE | wilcox | 25 | FALSE | both | 1000 | 50 | genesets.xlsx |

---

# 3 BridgeQC

## 3.1 Sample-level Characteristics and Quality Control Metrics

### 3.1.1 Metadata

| sample | tissue | tissue\_type | disease | age | sex | race | smoking | sample\_id |
| --- | --- | --- | --- | --- | --- | --- | --- | --- |
| acute-A-air | lung | small\_airway\_epithelial\_culture | control | 67 | female | caucasian | no | S1 |
| acute-A-smoke | lung | small\_airway\_epithelial\_culture | control | 67 | female | caucasian | no | S2 |
| acute-B-air | lung | small\_airway\_epithelial\_culture | control | 42 | female | hispanic | no | S3 |
| acute-B-smoke | lung | small\_airway\_epithelial\_culture | control | 42 | female | hispanic | no | S4 |
| acute-C-air | lung | small\_airway\_epithelial\_culture | control | 38 | male | caucasian | no | S5 |
| acute-C-smoke | lung | small\_airway\_epithelial\_culture | control | 38 | male | caucasian | no | S6 |
| acute-D-air | lung | small\_airway\_epithelial\_culture | copd | 57 | female | caucasian | yes | S7 |
| acute-D-smoke | lung | small\_airway\_epithelial\_culture | copd | 57 | female | caucasian | yes | S8 |
| acute-E-air | lung | small\_airway\_epithelial\_culture | copd | 51 | female | caucasian | yes | S9 |
| acute-E-smoke | lung | small\_airway\_epithelial\_culture | copd | 51 | female | caucasian | yes | S10 |
| acute-F-air | lung | small\_airway\_epithelial\_culture | copd | 62 | female | black | yes | S11 |
| acute-F-smoke | lung | small\_airway\_epithelial\_culture | copd | 62 | female | black | yes | S12 |

### 3.1.2 Cells & Genes (RNA)

| sample\_id | pre\_qc\_gene | pre\_qc\_cell | post\_qc\_gene | post\_qc\_cell |
| --- | --- | --- | --- | --- |
| S1 | 28,359 | 3,185 | 24,183 | 2,722 |
| S2 | 28,283 | 3,078 | 24,103 | 2,485 |
| S3 | 27,941 | 3,735 | 23,645 | 3,237 |
| S4 | 27,891 | 3,146 | 23,593 | 2,718 |
| S5 | 28,099 | 3,483 | 23,907 | 2,907 |
| S6 | 28,968 | 4,385 | 24,901 | 3,882 |
| S7 | 27,911 | 3,059 | 23,565 | 2,639 |
| S8 | 27,451 | 2,672 | 23,156 | 2,322 |
| S9 | 28,361 | 3,958 | 24,320 | 3,462 |
| S10 | 28,100 | 3,419 | 23,944 | 2,958 |
| S11 | 27,819 | 3,154 | 23,501 | 2,614 |
| S12 | 27,588 | 2,904 | 23,374 | 2,254 |

### 3.1.3 UMI per cell (RNA)

Vales are post-QC.

| sample\_id | min | 0% | 25% | 50% | 75% | 100% | max |
| --- | --- | --- | --- | --- | --- | --- | --- |
| S1 | 755 | 755 | 10,330 | 13,077 | 17,901 | 50,093 | 50,093 |
| S2 | 754 | 754 | 12,730 | 15,754 | 20,333 | 57,785 | 57,785 |
| S3 | 770 | 770 | 9,771 | 13,210 | 18,227 | 65,647 | 65,647 |
| S4 | 754 | 754 | 11,650 | 14,530 | 18,866 | 69,823 | 69,823 |
| S5 | 750 | 750 | 8,439 | 11,219 | 14,784 | 43,828 | 43,828 |
| S6 | 753 | 753 | 10,481 | 13,479 | 16,994 | 56,110 | 56,110 |
| S7 | 754 | 754 | 10,338 | 13,424 | 18,580 | 55,735 | 55,735 |
| S8 | 759 | 759 | 11,062 | 13,706 | 16,886 | 56,583 | 56,583 |
| S9 | 752 | 752 | 9,554 | 13,014 | 18,036 | 49,477 | 49,477 |
| S10 | 769 | 769 | 9,544 | 14,424 | 20,350 | 51,402 | 51,402 |
| S11 | 768 | 768 | 7,512 | 10,119 | 15,879 | 54,561 | 54,561 |
| S12 | 769 | 769 | 9,803 | 12,572 | 17,718 | 59,257 | 59,257 |

### 3.1.4 Gene per cell (RNA)

Vales are post-QC.

| sample\_id | min | 0% | 25% | 50% | 75% | 100% | max |
| --- | --- | --- | --- | --- | --- | --- | --- |
| S1 | 438 | 438 | 3,244 | 3,964 | 4,550 | 9,186 | 9,186 |
| S2 | 440 | 440 | 3,721 | 4,310 | 4,812 | 9,741 | 9,741 |
| S3 | 409 | 409 | 2,966 | 3,473 | 4,040 | 8,454 | 8,454 |
| S4 | 481 | 481 | 3,416 | 3,923 | 4,477 | 9,025 | 9,025 |
| S5 | 402 | 402 | 2,698 | 3,423 | 4,148 | 8,234 | 8,234 |
| S6 | 467 | 467 | 3,030 | 3,859 | 4,450 | 8,577 | 8,577 |
| S7 | 457 | 457 | 3,219 | 3,759 | 4,290 | 10,362 | 10,362 |
| S8 | 424 | 424 | 3,445 | 3,948 | 4,400 | 8,542 | 8,542 |
| S9 | 427 | 427 | 3,166 | 3,874 | 4,397 | 8,077 | 8,077 |
| S10 | 514 | 514 | 3,306 | 3,998 | 4,517 | 8,075 | 8,075 |
| S11 | 327 | 327 | 2,784 | 3,342 | 4,110 | 8,880 | 8,880 |
| S12 | 528 | 528 | 3,351 | 3,842 | 4,499 | 8,519 | 8,519 |

### 3.1.5 Mitochondrial (%) per cell (RNA)

Vales are post-QC.

| sample\_id | min | 0% | 25% | 50% | 75% | 100% | max |
| --- | --- | --- | --- | --- | --- | --- | --- |
| S1 | 0 | 0 | 4.83 | 6.98 | 8.29 | 14.97 | 14.97 |
| S2 | 0 | 0 | 4.78 | 6.15 | 7.30 | 15.00 | 15.00 |
| S3 | 0 | 0 | 2.62 | 4.18 | 6.18 | 14.95 | 14.95 |
| S4 | 0 | 0 | 3.61 | 5.50 | 6.88 | 14.75 | 14.75 |
| S5 | 0 | 0 | 3.80 | 6.05 | 7.58 | 15.00 | 15.00 |
| S6 | 0 | 0 | 4.30 | 6.19 | 7.40 | 14.83 | 14.83 |
| S7 | 0 | 0 | 3.84 | 5.99 | 7.13 | 14.95 | 14.95 |
| S8 | 0 | 0 | 6.24 | 7.52 | 8.67 | 14.93 | 14.93 |
| S9 | 0 | 0 | 3.45 | 5.22 | 6.73 | 14.96 | 14.96 |
| S10 | 0 | 0 | 4.32 | 5.70 | 7.40 | 15.00 | 15.00 |
| S11 | 0 | 0 | 4.19 | 5.92 | 7.68 | 15.00 | 15.00 |
| S12 | 0 | 0 | 4.94 | 6.51 | 8.60 | 14.95 | 14.95 |

## 

---

## 3.2 Distribution Plots for Quality Control Metrics

Thresholds, represented by dashed lines, were implemented to filter
the data and only retain cells of high quality.

### 3.2.1 UMI per cell

### 3.2.2 Gene per cell

### 3.2.3 Mitochondrial (%) per cell

## 

---

## 3.3 Barcodes contamination

### 3.3.1 Raw count

Number of barcodes shared between pairs of samples post-QC.

|  | S1 | S2 | S3 | S4 | S5 | S6 | S7 | S8 | S9 | S10 | S11 | S12 |
| --- | --- | --- | --- | --- | --- | --- | --- | --- | --- | --- | --- | --- |
| S1 | 2722 | 10 | 16 | 8 | 10 | 11 | 15 | 12 | 10 | 13 | 6 | 7 |
| S2 | 10 | 2485 | 11 | 5 | 14 | 12 | 11 | 5 | 12 | 10 | 6 | 12 |
| S3 | 16 | 11 | 3237 | 9 | 13 | 17 | 7 | 6 | 15 | 10 | 16 | 8 |
| S4 | 8 | 5 | 9 | 2718 | 7 | 14 | 9 | 6 | 16 | 8 | 8 | 9 |
| S5 | 10 | 14 | 13 | 7 | 2907 | 4 | 10 | 8 | 11 | 8 | 9 | 7 |
| S6 | 11 | 12 | 17 | 14 | 4 | 3882 | 20 | 10 | 22 | 11 | 15 | 10 |
| S7 | 15 | 11 | 7 | 9 | 10 | 20 | 2639 | 6 | 14 | 11 | 8 | 7 |
| S8 | 12 | 5 | 6 | 6 | 8 | 10 | 6 | 2322 | 11 | 10 | 6 | 2 |
| S9 | 10 | 12 | 15 | 16 | 11 | 22 | 14 | 11 | 3462 | 13 | 14 | 12 |
| S10 | 13 | 10 | 10 | 8 | 8 | 11 | 11 | 10 | 13 | 2958 | 7 | 9 |
| S11 | 6 | 6 | 16 | 8 | 9 | 15 | 8 | 6 | 14 | 7 | 2614 | 8 |
| S12 | 7 | 12 | 8 | 9 | 7 | 10 | 7 | 2 | 12 | 9 | 8 | 2254 |

### 3.3.2 Jaccard Index

Fraction (%) of barcodes shared between pairs of samples post-QC.

|  | S1 | S2 | S3 | S4 | S5 | S6 | S7 | S8 | S9 | S10 | S11 | S12 |
| --- | --- | --- | --- | --- | --- | --- | --- | --- | --- | --- | --- | --- |
| S1 | 100.00 | 0.38 | 0.54 | 0.29 | 0.36 | 0.33 | 0.56 | 0.48 | 0.32 | 0.46 | 0.22 | 0.28 |
| S2 | 0.38 | 100.00 | 0.38 | 0.19 | 0.52 | 0.38 | 0.43 | 0.21 | 0.40 | 0.37 | 0.24 | 0.51 |
| S3 | 0.54 | 0.38 | 100.00 | 0.30 | 0.42 | 0.48 | 0.24 | 0.22 | 0.45 | 0.32 | 0.55 | 0.29 |
| S4 | 0.29 | 0.19 | 0.30 | 100.00 | 0.25 | 0.42 | 0.34 | 0.24 | 0.52 | 0.28 | 0.30 | 0.36 |
| S5 | 0.36 | 0.52 | 0.42 | 0.25 | 100.00 | 0.12 | 0.36 | 0.31 | 0.35 | 0.27 | 0.33 | 0.27 |
| S6 | 0.33 | 0.38 | 0.48 | 0.42 | 0.12 | 100.00 | 0.61 | 0.32 | 0.60 | 0.32 | 0.46 | 0.33 |
| S7 | 0.56 | 0.43 | 0.24 | 0.34 | 0.36 | 0.61 | 100.00 | 0.24 | 0.46 | 0.39 | 0.30 | 0.29 |
| S8 | 0.48 | 0.21 | 0.22 | 0.24 | 0.31 | 0.32 | 0.24 | 100.00 | 0.38 | 0.38 | 0.24 | 0.09 |
| S9 | 0.32 | 0.40 | 0.45 | 0.52 | 0.35 | 0.60 | 0.46 | 0.38 | 100.00 | 0.40 | 0.46 | 0.42 |
| S10 | 0.46 | 0.37 | 0.32 | 0.28 | 0.27 | 0.32 | 0.39 | 0.38 | 0.40 | 100.00 | 0.25 | 0.35 |
| S11 | 0.22 | 0.24 | 0.55 | 0.30 | 0.33 | 0.46 | 0.30 | 0.24 | 0.46 | 0.25 | 100.00 | 0.33 |
| S12 | 0.28 | 0.51 | 0.29 | 0.36 | 0.27 | 0.33 | 0.29 | 0.09 | 0.42 | 0.35 | 0.33 | 100.00 |

## 

---

## 3.4 Doublet Cell Detection using Scrublet Scoring System

Observed scores are used for doublet classification. Dashed line
indicates the threshold used to identify doublets.

### 3.4.1 Observed scores

### 3.4.2 Simulated scores

## 

---

## 3.5 Impact of Quality Control on Cell, Gene, and UMI Abundances

### 3.5.1 Number of cells and genes per sample

diamonds and diamonds refer to before and after QC,
respectively.

### 3.5.2 Average number of UMIs/cell and genes/cell per sample

The error bars represent the standard deviation of the number of UMIs
and genes across cells per sample.

## 

---

## 3.6 Key QC Metrics of Merged Samples

|  | min | 0% | 25% | 50% | 75% | 100% | max |
| --- | --- | --- | --- | --- | --- | --- | --- |
| UMI per cell | 750 | 750 | 9,908 | 13,304 | 17,907 | 69,823 | 69,823 |
| Gene per cell | 327 | 327 | 3,144 | 3,818 | 4,420 | 10,362 | 10,362 |
| Mitochondrial (%) per cell | 0.00 | 0.00 | 4.02 | 5.97 | 7.51 | 15.00 | 15.00 |

---

## 3.7 Visualization of Merged scRNA-Seq Object in 2D Space

Note: Labels may have been removed if they overlap excessively.

### 3.7.1 Signacx SPRING Projection

### 3.7.2 Seurat UMAP Projection

### 3.7.3 Seurat TSNE Projection

### 3.7.4 Seurat UMAP Projection (no-batch correction)

### 3.7.5 Seurat TSNE Projection (no-batch correction)

## 

---

## 3.8 Visualizing Cell QC Metrics Using Dimensionality Reduction

Note: QC metrics based on kernel density estimation.

### 3.8.1 UMI per cell - SPRING Projection

### 3.8.2 Gene per cell - SPRING Projection

### 3.8.3 Mitochondrial (%) per cell - SPRING Projection

### 3.8.4 UMI per cell - UMAP Projection

### 3.8.5 Gene per cell - UMAP Projection

### 3.8.6 Mitochondrial (%) per cell - UMAP Projection

### 3.8.7 UMI per cell - TSNE Projection

### 3.8.8 Gene per cell - TSNE Projection

### 3.8.9 Mitochondrial (%) per cell - TSNE Projection

## 

---

# 4 BridgeCluster

Note: Labels may have been removed if they overlap excessively.

## 4.1 Visualizing Cell Populations with Dimensionality Reduction

### 4.1.1 Signacx SPRING Projection

### 4.1.2 Seurat UMAP Projection

### 4.1.3 Seurat TSNE Projection

## 

---

## 4.2 Cell Cluster Composition

Values at the top of each bar indicate the percentage of cells

### 4.2.1 Seurat

### 4.2.2 Signacx

## 

---

## 4.3 Statistical Dispersion Analysis of Cell Composition in Clusters across sample

This measurement is a proxy to batch effect artifacts. Values
adjacent to each point indicate the number of cells.

### 4.3.1 Seurat

### 4.3.2 Signacx

## 

---

## 4.4 Markers per cluster

Top 25 differentially expressed genes ( p\_val\_adj < 0.05 and pct.1
> 0.5 ) for each of the clusters

### 4.4.1 Seurat

### 4.4.2 Signacx

## 

---

# 5 BridgeAnnotation

## 5.1 Visualizing Distinct Cell Type Populations with Dimensionality Reduction

Note: Labels may have been removed if they overlap excessively.

### 5.1.1 Signacx SPRING Projection

### 5.1.2 Sargent SPRING Projection

### 5.1.3 Signacx UMAP Projection

### 5.1.4 Sargent UMAP Projection

### 5.1.5 Signacx TSNE Projection

### 5.1.6 Sargent TSNE Projection

## 

---

## 5.2 Cell Type Population Composition

Values at the top of each bar indicate the percentage of cells

### 5.2.1 Signacx

### 5.2.2 Sargent

## 

---

## 5.3 Visualizing the Hierarchy of User-Provided Gene Sets

---

## 5.4 Evaluating Sargent Cell Type Annotation with Gene Marker Expression

Expression level of selected genes for each group. Color indicates
the average expression and size indicates the percent expressed.

### 5.4.1 Master-1

### 5.4.2 Master-2

### 5.4.3 Master-3

### 5.4.4 EPITHELIAL

## 

---

# 6 BridgeTrajectory

## 6.1 Visualizing Inferred Trajectory on Manifolds

Note: Labels may have been removed if they overlap excessively.

### 6.1.1 Seurat UMAP

### 6.1.2 Seurat TSNE

## 

---

## 6.2 Visualizing Inferred Trajectory of ‘pseudotime’ Across Different ‘lineages’ and ‘clusters’

### 6.2.1 Seurat UMAP

### 6.2.2 Seurat TSNE

## 

---

## 6.3 Trajectory-based Differential Expression Analysis

Top 50 differentially expressed genes (FDR < 0.05) along each
inferred lineage.

### 6.3.1 Seurat UMAP

### 6.3.2 Seurat TSNE

## 

---

# 7 References

Publication: paper

Data Availability: data

---

# 8 Session Information

This is the output of `sessionInfo()` on the computing
system on which this document was compiled

```
## R version 4.3.2 (2023-10-31)
## Platform: x86_64-pc-linux-gnu (64-bit)
## Running under: Ubuntu 20.04.6 LTS
## 
## Matrix products: default
## BLAS:   /usr/lib/x86_64-linux-gnu/blas/libblas.so.3.9.0 
## LAPACK: /usr/lib/x86_64-linux-gnu/lapack/liblapack.so.3.9.0
## 
## Random number generation:
##  RNG:     L'Ecuyer-CMRG 
##  Normal:  Inversion 
##  Sample:  Rejection 
##  
## locale:
## [1] C
## 
## time zone: Etc/UTC
## tzcode source: system (glibc)
## 
## attached base packages:
## [1] stats4    grid      stats     graphics  grDevices
## [6] utils     datasets  methods   base     
## 
## other attached packages:
##  [1] edgeR_4.0.2                 limma_3.58.1               
##  [3] slingshot_2.10.0            TrajectoryUtils_1.10.0     
##  [5] SingleCellExperiment_1.24.0 SummarizedExperiment_1.32.0
##  [7] Biobase_2.62.0              GenomicRanges_1.54.1       
##  [9] GenomeInfoDb_1.38.1         IRanges_2.36.0             
## [11] S4Vectors_0.40.1            BiocGenerics_0.48.1        
## [13] MatrixGenerics_1.14.0       matrixStats_1.1.0          
## [15] princurve_2.1.6             Nebulosa_1.12.0            
## [17] patchwork_1.1.3             data.table_1.14.8          
## [19] ComplexHeatmap_2.18.0       visNetwork_2.1.2           
## [21] plotly_4.10.3               data.tree_1.1.0            
## [23] DT_0.30                     readxl_1.4.3               
## [25] gtools_3.9.5                gplots_3.1.3               
## [27] gridtext_0.1.5              igraph_1.5.1               
## [29] sargent_1.0.1               SignacX_2.2.0              
## [31] harmony_1.2.0               Rcpp_1.0.11                
## [33] kableExtra_1.3.4            purrr_1.0.2                
## [35] reticulate_1.34.0           RColorBrewer_1.1-3         
## [37] cowplot_1.1.1               gridExtra_2.3              
## [39] pheatmap_1.0.12             ggrepel_0.9.4              
## [41] ggplot2_3.4.4               Seurat_5.0.1               
## [43] SeuratObject_5.0.1          sp_2.1-1                   
## [45] dplyr_1.1.4                 optparse_1.7.3             
## 
## loaded via a namespace (and not attached):
##   [1] spatstat.sparse_3.0-3     bitops_1.0-7             
##   [3] httr_1.4.7                webshot_0.5.5            
##   [5] doParallel_1.0.17         tools_4.3.2              
##   [7] sctransform_0.4.1         utf8_1.2.4               
##   [9] R6_2.5.1                  mgcv_1.9-0               
##  [11] lazyeval_0.2.2            uwot_0.1.16              
##  [13] ggdist_3.3.0              GetoptLong_1.0.5         
##  [15] withr_2.5.2               progressr_0.14.0         
##  [17] cli_3.6.1                 spatstat.explore_3.2-5   
##  [19] fastDummies_1.7.3         labeling_0.4.3           
##  [21] sass_0.4.7                mvtnorm_1.2-3            
##  [23] spatstat.data_3.0-3       proxy_0.4-27             
##  [25] ggridges_0.5.4            pbapply_1.7-2            
##  [27] commonmark_1.9.0          systemfonts_1.0.5        
##  [29] svglite_2.1.2             parallelly_1.36.0        
##  [31] rstudioapi_0.15.0         generics_0.1.3           
##  [33] shape_1.4.6               crosstalk_1.2.1          
##  [35] ica_1.0-3                 spatstat.random_3.2-1    
##  [37] distributional_0.3.2      Matrix_1.6-3             
##  [39] fansi_1.0.5               DescTools_0.99.51        
##  [41] abind_1.4-5               lifecycle_1.0.4          
##  [43] yaml_2.3.7                SparseArray_1.2.2        
##  [45] Rtsne_0.16                promises_1.2.1           
##  [47] crayon_1.5.2              miniUI_0.1.1.1           
##  [49] lattice_0.22-5            pillar_1.9.0             
##  [51] knitr_1.45                rjson_0.2.21             
##  [53] boot_1.3-28.1             gld_2.6.6                
##  [55] future.apply_1.11.0       codetools_0.2-19         
##  [57] leiden_0.4.3.1            glue_1.6.2               
##  [59] vctrs_0.6.4               png_0.1-8                
##  [61] spam_2.10-0               neuralnet_1.44.2         
##  [63] cellranger_1.1.0          gtable_0.3.4             
##  [65] cachem_1.0.8              ks_1.14.1                
##  [67] xfun_0.41                 S4Arrays_1.2.0           
##  [69] mime_0.12                 pracma_2.4.4             
##  [71] survival_3.5-7            pbmcapply_1.5.1          
##  [73] iterators_1.0.14          statmod_1.5.0            
##  [75] ellipsis_0.3.2            fitdistrplus_1.1-11      
##  [77] ROCR_1.0-11               nlme_3.1-163             
##  [79] bit64_4.0.5               RcppAnnoy_0.0.21         
##  [81] bslib_0.6.0               irlba_2.3.5.1            
##  [83] KernSmooth_2.23-22        colorspace_2.1-0         
##  [85] Exact_3.2                 tidyselect_1.2.0         
##  [87] bit_4.0.5                 compiler_4.3.2           
##  [89] rvest_1.0.3               hdf5r_1.3.8              
##  [91] expm_0.999-7              xml2_1.3.5               
##  [93] DelayedArray_0.28.0       scales_1.2.1             
##  [95] caTools_1.18.2            lmtest_0.9-40            
##  [97] stringr_1.5.1             digest_0.6.33            
##  [99] goftest_1.2-3             presto_1.0.0             
## [101] spatstat.utils_3.0-4      rmarkdown_2.25           
## [103] RhpcBLASctl_0.23-42       XVector_0.42.0           
## [105] htmltools_0.5.7           pkgconfig_2.0.3          
## [107] sparseMatrixStats_1.14.0  highr_0.10               
## [109] fastmap_1.1.1             rlang_1.1.2              
## [111] GlobalOptions_0.1.2       htmlwidgets_1.6.3        
## [113] DelayedMatrixStats_1.24.0 shiny_1.8.0              
## [115] jquerylib_0.1.4           farver_2.1.1             
## [117] zoo_1.8-12                jsonlite_1.8.7           
## [119] mclust_6.0.1              RCurl_1.98-1.13          
## [121] magrittr_2.0.3            GenomeInfoDbData_1.2.11  
## [123] dotCall64_1.1-0           munsell_0.5.0            
## [125] stringi_1.8.2             rootSolve_1.8.2.4        
## [127] zlibbioc_1.48.0           MASS_7.3-60              
## [129] plyr_1.8.9                parallel_4.3.2           
## [131] listenv_0.9.0             lmom_3.0                 
## [133] deldir_2.0-2              splines_4.3.2            
## [135] tensor_1.5                circlize_0.4.15          
## [137] locfit_1.5-9.8            spatstat.geom_3.2-7      
## [139] markdown_1.11             RcppHNSW_0.5.0           
## [141] reshape2_1.4.4            evaluate_0.23            
## [143] foreach_1.5.2             httpuv_1.6.12            
## [145] RANN_2.6.1                tidyr_1.3.0              
## [147] getopt_1.20.4             polyclip_1.10-6          
## [149] future_1.33.0             clue_0.3-65              
## [151] scattermore_1.2           xtable_1.8-4             
## [153] e1071_1.7-13              RSpectra_0.16-1          
## [155] later_1.3.1               viridisLite_0.4.2        
## [157] class_7.3-22              tibble_3.2.1             
## [159] cluster_2.1.4             globals_0.16.2
```
